# Optimization of Drug Delivery by Drug-Eluting Stents

**DOI:** 10.1371/journal.pone.0130182

**Published:** 2015-06-17

**Authors:** Franz Bozsak, David Gonzalez-Rodriguez, Zachary Sternberger, Paul Belitz, Thomas Bewley, Jean-Marc Chomaz, Abdul I. Barakat

**Affiliations:** 1 Laboratoire d’Hydrodynamique (LadHyX), École Polytechnique—CNRS, Palaiseau cedex, France; 2 UCSD Flow Control and Coordinated Robotics Labs Dept of MAE, UC San Diego, La Jolla, CA, USA; University of California Berkeley, UNITED STATES

## Abstract

Drug-eluting stents (DES), which release anti-proliferative drugs into the arterial wall in a controlled manner, have drastically reduced the rate of in-stent restenosis and revolutionized the treatment of atherosclerosis. However, late stent thrombosis remains a safety concern in DES, mainly due to delayed healing of the endothelial wound inflicted during DES implantation. We present a framework to optimize DES design such that restenosis is inhibited without affecting the endothelial healing process. To this end, we have developed a computational model of fluid flow and drug transport in stented arteries and have used this model to establish a metric for quantifying DES performance. The model takes into account the multi-layered structure of the arterial wall and incorporates a reversible binding model to describe drug interaction with the cells of the arterial wall. The model is coupled to a novel optimization algorithm that allows identification of optimal DES designs. We show that optimizing the period of drug release from DES and the initial drug concentration within the coating has a drastic effect on DES performance. Paclitaxel-eluting stents perform optimally by releasing their drug either very rapidly (within a few hours) or very slowly (over periods of several months up to one year) at concentrations considerably lower than current DES. In contrast, sirolimus-eluting stents perform optimally only when drug release is slow. The results offer explanations for recent trends in the development of DES and demonstrate the potential for large improvements in DES design relative to the current state of commercial devices.

## Introduction

Drug-eluting stents (DES) have proven highly effective in reducing restenosis rates relative to bare metal stents. However, a persistent concern associated with the use of DES is late stent thrombosis, which can occur up to several years after stent implantation [1–3]. Although its development remains incompletely understood, late stent thrombosis is thought to occur as a result of the delayed healing of the endothelium following its denudation by both the stent and the balloon upon which the stent is typically deployed. In support of this notion, a recent study has demonstrated that DES can in some cases remain unendothelialized five years after stent deployment [1]. In contrast, bare metal stents are typically covered with new endothelium within six months of the stenting procedure [4, 5].

Drugs eluted from DES, most commonly paclitaxel or sirolimus, arrest the proliferation of smooth muscle cells (SMCs) in the arterial wall and hence inhibit vascular restenosis. A likely reason for the delayed endothelial healing in the case of DES is that these same drugs also inhibit endothelial cell (EC) proliferation and migration [6–10] and thus greatly limit endothelial wound healing. A key question that arises in the design of DES is whether or not it is possible to deliver anti-proliferative drugs at sufficiently high concentrations to SMCs to prevent restenosis while simultaneously maintaining a sufficiently low drug concentration at the EC surface to allow sufficiently rapid endothelial wound closure.

We recently developed a computational model for the transport of drugs eluted from DES within the arterial wall [11]. The model considered the arterial wall to consist of a two porous layers representing the subendothelial intima and the media. Drug release from the stent was assumed to occur by diffusion, and drug transport in the arterial wall was assumed to occur by convection and diffusion with the drug also undergoing a reversible reaction in the media to represent its binding to SMCs. The baseline model assumed a completely denuded endothelium in the stented portion of the artery, while the endothelium remained intact both upstream and downstream of the stent. The model was applied to the transport of both paclitaxel and sirolimus, and the results revealed important differences between the two drugs in transport characteristics and dynamics. Importantly, the results suggested that drug distribution within the arterial wall depends on a number of parameters including the drug, its release rate into the arterial wall, and its initial concentration in the stent polymer. These findings serve as the primary motivation for the present study which focuses on the optimization of drug delivery strategies from DES for both paclitaxel and sirolimus.

The notion of using optimization in stent design and performance assessment has previously been invoked in other contexts. For instance, previous studies have reported the optimization of stent strut geometry with the goal of minimizing blood flow disturbance in the arterial lumen [12–15], stresses in the stent itself [16], or stresses in the arterial wall [17]. Pant and collaborators recently reported the first attempt at including multiple design objectives and multiple physical phenomena in the optimization process with the use of a steady (time-independent) transport model to investigate the effect of DES geometric design on the homogeneity of drug distribution and its average concentration in the arterial wall [18, 19]. Coupling a corrosion model to a global optimization strategy [20] Grogan et. al [21] have optimized the geometric design of the bioresorbable AMS stent (made by Biotronik) to optimize its degradation performance. In all previous studies, stent geometry served as the design variable. We follow a different strategy and seek to optimize drug release kinetics (elution process) from the polymer coating as well as the drug concentration initially loaded onto this coating. Release kinetics have been at the heart of experimental [22–26] and computational [27, 28] investigations over the past few years. However, optimal release kinetics remain an open question in the design process of DES [29, 30]. Optimizing the delivery process of the eluted drug holds the promise of providing strategies that at least in part address the problem of delayed endothelial healing.

In the present study, we develop a strategy to identify optimal drug delivery by DES. The overall goal of the optimization is to maintain an efficacious but sub-toxic drug concentration in the arterial media while simultaneously targeting minimal drug concentration at the endothelial surface in order to allow stent re-endothelialization. The design variables are assumed to be the initial drug concentration loaded onto the stent and the drug release rate from the stent coating. The optimization is implemented by coupling a novel Surrogate Management Framework (SMF) optimization algorithm [31, 32] with our physiologically-based computational model of drug transport in stented arteries (see [11]). This approach is applied to determine optimal drug delivery strategies for both paclitaxel- and sirolimus-eluting stents. The results suggest that optimal drug delivery strategies for paclitaxel are very different from those for sirolimus and that today’s commercial stents are far from optimal.

## Materials and Methods

### Computational model

#### Modeling drug transport and reaction in the arterial wall

We have recently described a computational model for the transport and reaction within the arterial wall of drugs released by DES [11]. In the present work, we use this model to optimize the elution of the two small hydrophobic drugs paclitaxel and sirolimus from DES. Because the model has been described in detail in our previous work [11], we will only briefly overview it here and highlight its most important features.

**Fig 1:**
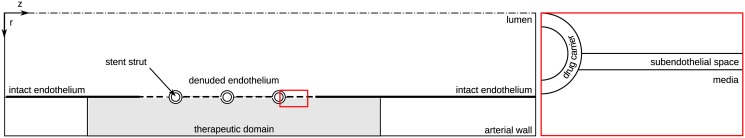
The computational model used in the simulations considers the endothelium to be denuded upstream and downstream of the stent, over a distance that is one half of the inter-strut spacing measured from the stent strut centers. The subendothelial space and the media are modeled as distinct layers of the arterial wall.

As depicted in Fig 1, the model considers a straight and axisymmetric arterial segment. The arterial wall is assumed rigid and is modeled as a two-layered structure with the subendothelial space (SES) of the intima and the media represented as distinct porous layers. The endothelium and internal elastic lamina (IEL) are considered as interfacial matching conditions, while the adventitia is modeled as a boundary condition of the media. The model uses a recently developed reversible second-order reaction model [33] to account for the interaction of the drug with the cells of the arterial wall. The DES is assumed to consist of three circular cross-section struts spaced at intervals of 0.7 mm. Each strut has a diameter of 100 μm and is covered with a 10 μm-thick polymer coating, reflecting approximate dimensions of typical second-generation DES [34]. We assume that as a result of stent deployment, the endothelium is completely denuded within the stented portion of the vessel as well as up to a distance equal to half of the inter-strut spacing (0.35 mm) both upstream and downstream of the stent but is intact otherwise. We define the *therapeutic domain* as the volume of the arterial wall containing the stent and extending by two-thirds of the stent length both upstream and downstream of the stent (Fig 1). The flow in the arterial lumen is governed by the Navier-Stokes equations, while flow in the arterial wall is assumed to be governed by Darcy’s Law. The entire flow field is treated as steady. Time-dependent drug transport occurs as follows: 1) in the lumen the drug is transported via convection and diffusion; 2) in the polymer coating of the stent drug transport is assumed to be purely diffusive; 3) in the SES and media drug transport is via convection and diffusion with both specific binding and unbinding of the drug to cells of the arterial wall (ECs and SMCs) and non-specific binding to the extracellular matrix.

#### Modeling atherosclerotic arteries

Early atherosclerosis involves EC inflammation and intimal thickening [35, 36]. Whereas the healthy SES is virtually free of SMCs, the onset of atherosclerosis compromises the IEL and leads to SMC migration from the media into the SES. Our previous modeling [11] did not take the presence of SMCs in the SES into account. We wish to do so here by incorporating a drug reaction term in the SES so that drug transport in this layer is governed by the following averaged transport equation:

$$\begin{array}{c} \frac{\partial c_{\text{ses}}}{\partial t}+\Lambda_{\text{ses}}{\vec{u}}_{\text{ses}}\nabla c_{\text{ses}}=\nabla ({\vec{D}}_{\text{ses}}\nabla c_{\text{ses}})+R_{\text{ses}}\;, \end{array}$$ (1)

where *c*_ses_ is the superficially-averaged free concentration in the SES (averaging over both phases of the porous medium containing the pore space and the solid tissue space) and ${\vec{u}}_{\text{ses}}$ is the the average fluid velocity in the total volume (matrix plus pores). For the reaction term in the SES we assume a lower drug maximum binding site density *b*_max,ses_ compared to the maximum binding site density in the media *b*_max_. Moroever, transport of the drug through the SES is more severely hindered by the presence of SMCs resulting in an altered lag coefficient $\Lambda_{\text{ses}}=\frac{\gamma_{\text{ses}}}{{ɛ}_{\text{ses}}}$, porosity *ɛ*_ses_ and effective diffusivity ${\vec{D}}_{\text{ses}}$ of the drug in the SES relative to the case of an SMC-free SES. The reaction term in the SES for the bound drug *b*_ses_ is assumed to take the following form [11, 33]:

$$\begin{array}{c} -R_{\text{ses}}=\frac{\partial b_{\text{ses}}}{\partial t}={\left(1-\varepsilon_{\text{ses}}\right)}^{-1}\left(k_{f}c_{\text{ses}}(b_{\text{max},\text{ses}}-b_{\text{ses}})-k_{r}b_{\text{ses}}\right)\;, \end{array}$$ (2)

whereby the maximum binding site density in the SES *b*_max,ses_ is calculated as a fraction *ξ* of the maximum binding site density in the media *b*_max_. *ξ* represents the ratio of the volume fraction of SMCs in the SES to the volume fraction of SMCs in the media and is an adjustable parameter in the model. *k*_f_ and *k*_r_ are the drug binding rate coefficient and the drug unbinding rate constant, respectively.

To model the fact that the IEL becomes damaged as atherosclerosis progresses, we replace the Kedem-Katchalsky interface condition describing concentration and pressure discontinuity as used in the original model by a continuous formulation. To obtain the altered transport parameters (Λ_ses_, *ɛ*_ses_ and ${\vec{D}}_{\text{ses}}$), we apply the method proposed in [37] to the parameter values used in the non-diseased baseline case as detailed in Appendix 2. In order to allow direct comparisons between the baseline model and the case of a diseased vessel, we do not change the thickness of the SES layer; we assume that stent implantation compresses the diseased intima to the same extent as the healthy one.

#### Numerical methods

The governing equations are discretized by means of the finite element method using the commercial software package COMSOL Multiphysics 4.3a (COMSOL AB, Burlington, MA, USA). The tolerance threshold for the relative error of the solution (relative tolerance) of the momentum equations was set to 10^−9^. An analysis of the transport problem showed no change of the solution below a combination of relative tolerance of 10^−3^ and absolute tolerance of 10^−4^. The time advancing scheme is a backward differentiation formulation with variable order and time step size [38]. The maximum time step size is restricted to 1 hour (h). Reducing the maximum time step to one eighth of an hour did not change the solution, validating our choice for the maximum step size.

The mixed triangular and quadrilateral mesh is enhanced with boundary layer elements at the interface between the lumen and the arterial wall and at the interface of the stent polymer coating with the arterial wall. To smoothen the sharp initial condition from the stent polymer to the surrounding domain, the inner boundary of the triangular polymer mesh is enhanced with boundary layer elements, with the initial condition transitioning from *c*(*t* = 0) = 1 to *c*(*t* = 0) = 0 using an infinitely differentiable step function. The classical approach of a mesh independence study [39] was used to determine the number of elements in the lumen, the SES and in the polymer. More specifically, we successively increased the number of mesh elements in each of these layers by a factor of 1.5 to 2 until the time evolution of the average concentration in the SES and the polymer showed a relative difference of less than 1% from one mesh iteration to another. Similarly, we used the average wall shear stress along the lumen-wall interface and the flow profile downstream of the stent as the test quantities to verify grid independence in the lumen. In the media, however, the maximum cell size was limited by the occurrence of spurious oscillations in the solution. This resulted in an overall very fine mesh with approximately 290 000 elements. Computation time for one simulation performed on 2 cores of an Intel Xeon CPU X5680 @ 3.33GHz processor was approximately 2 h.

#### Time scales and dimensionless quantities for drug transport

In order to better interpret the results, it is useful to recall dimensionless quantities characterizing the transport problem (see [11] for details):

- Peclet number (Pe): ratio of diffusion time scale to convection time scale,
- Damköhler number (Da): ratio of diffusion time scale to binding time scale,
- and the ratio of the former two dimensionless quantities $\frac{\text{Da}}{\text{Pe}}$: ratio of convection time scale to binding time scale.

The values of these parameters used in the current simulations are given in Table 1 for both paclitaxel and sirolimus. A time scale that is not accounted for by these dimensionless quantities is the drug unbinding time scale, which is also shown in Table 1.

**Table 1:** Drug unbinding time scale and dimensionless quantities for the stented part of the media for paclitaxel and sirolimus.

Drug	Unbinding time (h)	Peclet number	Damköhler number	$\frac{\text{Damköhler number}}{\text{Peclet number}}$
Paclitaxel	210.7	13.0	6.75	0.5
Sirolimus	11.2	3.7	125.0	34

The major difference between the two drugs lies in the relative importance of convection compared to reaction: sirolimus’ binding rate is so high that it even dominates the convective transport ($\frac{\text{Da}}{\text{Pe}}\gg 1$), whereas paclitaxel is more sensitive to convection ($\frac{\text{Da}}{\text{Pe}}<1$). We can also see that drug unbinding from binding sites in the media occurs significantly more slowly for paclitaxel than for sirolimus. For both drugs, the unbinding time scale is the slowest time scale in the transport problem.

### Optimization formulation and methodology

#### Cost function

We formulate a cost function that needs to be minimized for optimal drug delivery from DES. Minimization of the cost function serves to accomplish the following two principal objectives:

1. *Therapeutically efficacious but sub-toxic drug concentration in the media*: The eluted drug needs to have its desired therapeutic effect. We assume that as long as the local drug concentration in the media remains within the drug’s therapeutic window, i.e. above an efficacious minimum threshold and below a toxic maximum concentration, the drug effectively arrests SMC proliferation thereby having the desired effect of preventing neointimal hyperplasia and vascular restenosis.
2. *Minimal drug concentration at the arterial wall luminal surface*: We need to minimize drug concentration at the luminal surface in order to allow endothelial wound healing to occur. We postulate that if the drug concentration at the luminal surface remains below the lower limit of the drug’s therapeutic window, then EC proliferation will be unhindered.

How well a particular stent design performs in accomplishing the two objectives described above can serve as a metric of the quality of the design. For the purpose of the current investigation, a *design* is defined by the two parameters that determine the drug delivery strategy from DES: the initial drug concentration *c*_0_ in the stent polymer coating and the characteristic drug release time from this polymer coating. Because drug release is assumed to occur by diffusion only, this characteristic time is given by $t_{\text{E}}=\frac{\pi L_{\text{c}}^{2}}{\left(4D_{\text{c}}\right)}$ [40, 41], where *L*_c_ is the thickness of the polymer coating and *D*_c_ is the drug diffusion coefficient in the coating. The two parameters defining a particular design serve as input for a simulation using the computational model described in the previous section. The resulting concentration distribution is then used as the basis to quantify the performance of a particular design by means of the following cost function:

$$\begin{array}{c} J_{\text{ETB}}\left(c_{0},t_{E}\right)={\bar{I}}_{m}+\frac{1}{3}\left({\bar{T}}_{l}+{\bar{T}}_{\text{ses}}+{\bar{T}}_{m}\right)+{\bar{B}}_{m}\;. \end{array}$$ (3)

**Fig 2:**
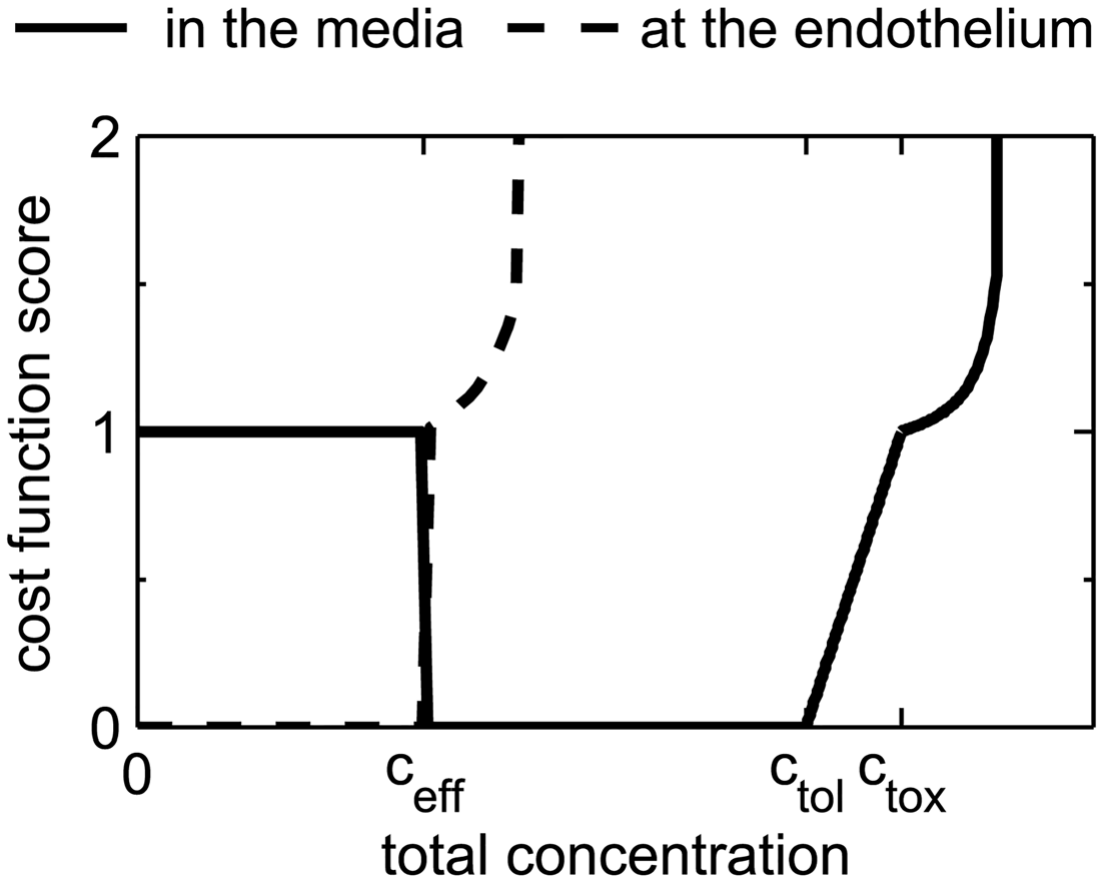
Shape of the score sheet for the cost function at the endothelium (dashed line) and in the media (continuous line).

In Eq (3), the cost function *J*_ETB_ is formulated as the sum of three scores evaluated within the therapeutic domain of the numerical model: a score that denotes drug inefficacy in the media (${\bar{I}}_{\text{m}}$), an overall toxicity score that consists of the arithmetic average of the three toxicity scores in the lumen, subendothelial space (SES), and media (${\bar{T}}_{\text{l}}$, ${\bar{T}}_{\text{ses}}$ and ${\bar{T}}_{\text{m}}$, respectively), and a buffer score (${\bar{B}}_{\text{m}}$). The shape of the resulting score sheet of the cost function *J*_ETB_ is schematically depicted in Fig 2 (solid line). We will now describe the three scores that constitute the score sheet of the cost function in detail.

The *inefficacy score* in the media *I*_m_ is defined as follows:

$$\begin{array}{c} I_{m}=\frac{1}{V_{m,\text{th}}}\int_{V_{m,\text{th}}}\varphi \;dV\;\text{where}\;\varphi =\{\begin{array}{cc} 1, & \text{if}\;c_{T,m}\leq c_{\text{eff}},\\ 0, & \text{otherwise}. \end{array} \end{array}$$ (4)

Every point in the medial portion of the therapeutic domain with a drug concentration below the minimum efficacious threshold *c*_eff_ is assigned a score of 1 and a score of 0 otherwise. The inefficacy score *I*_m_ is obtained by integrating the point-by-point scores over the entire medial therapeutic domain volume (*V*_m,th_) and then dividing by this volume. Therefore, *I*_m_ represents the percentage of the medial volume within the therapeutic domain where the total drug concentration *c*_T,m_ (i.e. sum of free and bound drug) falls below the minimum efficacy threshold. Eq (4) is evaluated for every time point of the simulation and then averaged over the entire simulation time *t*_end_ yielding the time-averaged inefficacy score ${\bar{I}}_{\text{m}}$. The simulation time *t*_end_ corresponds to the period of time over which the optimization is performed.

While the minimum efficacious concentration *c*_eff_ sets the lower limit of the drug therapeutic window, the toxic concentration *c*_tox_ defines the upper limit. In the media, the *toxicity score* ${\bar{T}}_{\text{m}}$ is obtained through the following series of steps. First, similar to the inefficacy parameter, the expression

$$\begin{array}{c} \vartheta_{m}=\frac{1}{V_{m,\text{th}}}\int_{V_{m,\text{th}}}\vartheta \;dV\;\text{where}\;\vartheta =\{\begin{array}{cc} \frac{c_{T,m}-c_{\text{tox}}}{\gamma_{\text{thr}}c_{\text{tox}}}, & \text{if}\;c_{T,m}\geq c_{\text{tox}},\\ 0, & \text{otherwise}. \end{array} \end{array}$$ (5)

is evaluated. This integral quantifies the fractional volume of the media within the therapeutic domain that is exposed to toxic drug concentrations, with the score weighted by the relative deviation from the toxic threshold so that the higher the concentration above the toxic threshold, the higher the score (and hence the larger the penalty). The scaling factor *γ*_thr_ provides a mechanism for adjusting the weighting to establish which deviation from the toxic threshold is considered equally harmful as a concentration that is below the efficacious threshold (i.e. a score of 1). Once this first step is completed, the second step consists of mapping the result of Eq (5) onto a hyperbola whose asymptote is at the limit value *ϑ*_lim_:

$$\begin{array}{c} T_{m}=\{\begin{array}{cc} 1+\frac{\vartheta_{\text{lim}}-\vartheta_{\text{thr}}}{\vartheta_{\text{lim}}-\vartheta_{m}}\frac{\vartheta_{m}}{\vartheta_{\text{thr}}}, & \text{if}\;0\leq \vartheta_{m}<\vartheta_{\text{lim}},\\ \infty , & \text{otherwise}. \end{array} \end{array}$$ (6)

The slope of this hyperbola is adjusted such that it passes through 1 at the threshold value *ϑ*_thr_. Furthermore, the hyperbola is lifted by 1 to emphasize that any toxic concentration is undesirable. The rapid increase in toxicity score as drug concentrations increase above the toxic threshold underscores the notion that these concentrations should be avoided by all means and serves to drive the optimization away from these concentrations.

The arterial wall luminal surface is handled virtually identically to the media with the following two equations:

$$\begin{array}{c} \vartheta_{S,l}=\frac{1}{S_{l,\text{th}}}\int_{S_{l,\text{th}}}\vartheta_{S}\;dS\;\text{where}\;\vartheta_{S}=\{\begin{array}{cc} \frac{c_{l}-c_{\text{eff}}}{\gamma_{\text{thr}}c_{\text{eff}}}, & \text{if}\;c_{l}\geq c_{\text{eff}},\\ 0, & \text{otherwise}. \end{array} \end{array}$$ (7)

$$\begin{array}{c} T_{l}=\{\begin{array}{cc} 1+\frac{\vartheta_{\text{lim}}-\vartheta_{\text{thr}}}{\vartheta_{\text{lim}}-\vartheta_{S,l}}\frac{\vartheta_{S,l}}{\vartheta_{\text{thr}}}, & \text{if}\;0\leq \vartheta_{S,l}<\vartheta_{\text{lim}},\\ \infty , & \text{otherwise}. \end{array} \end{array}$$ (8)

There are two differences to point out: 1) Given that we are now considering the lumen-wall interface, the integral in Eq (7) becomes a surface integral rather than a volume integral. 2) At the luminal surface we do not want the drug to impair EC proliferation; therefore, we consider that the “toxic” concentration limit is the minimum efficacious concentration and formulate the toxicity score in a manner to drive the algorithm towards concentrations lower than this threshold. We note that because a concentration jump occurs across the luminal surface whenever endothelium is present, we evaluate the interfacial toxicity parameter from both the luminal side (denoted by *ϑ*_S,l_ and *T*_l_) and the SES side (denoted *ϑ*_S,ses_ and *T*_ses_). The SES evaluations are similar to those shown above for the lumen but with the concentration *c*_l_ replaced by *c*_ses_. The arithmetic mean of the toxicity scores from the luminal and SES sides provides the toxicity score of the luminal surface *T*_e_. As in the case of the inefficacy score, the toxicity scores are evaluated for every time point of the simulation and then averaged over the entire simulation time *t*_end_. The resulting score sheet of the cost function at the luminal surface is schematically depicted in Fig 2 (dashed line).

The final term appearing in the cost function is the *buffer score* *B*_m_ which is defined as follows:

$$\begin{array}{c} B_{m}=\frac{1}{V_{m,t}}\int_{V_{m,t}}\beta \;dV\;\text{where}\;\beta =\{\begin{array}{cc} \frac{c_{T,m}-c_{\text{tol}}}{c_{\text{tox}}-c_{\text{tol}}}, & \text{if}\;c_{\text{tol}}\leq c_{T,m}<c_{\text{tox}},\\ 0, & \text{otherwise}. \end{array} \end{array}$$ (9)

The portion of the therapeutic domain of the media with a concentration superior to a tolerable value *c*_tol_ but inferior to the toxic threshold *c*_tox_ is weighted on a scale that increases linearly from 0 to 1 as the drug concentration approaches *c*_tox_. The spatially-averaged *B*_m_ is then averaged over the simulation period *t*_end_ to form the final buffer score ${\bar{B}}_{\text{m}}$.

The primary purpose of ${\bar{B}}_{\text{m}}$ is to create a buffer region in the optimization by penalizing concentrations close to the toxic limit *c*_tox_. The value of *c*_tol_ ensures a certain robustness of the optimization, in the sense that optima that are immediately adjacent to non-optimal regions can be avoided. In addition to this role, the tolerable concentration *c*_tol_ also drives the optimization towards designs with a more spatially homogeneous drug distribution in the arterial wall. Designs that lead to concentrations that lie within the reduced concentration window bound by *c*_eff_ and *c*_tol_ have a smaller concentration variation and are thus more homogeneous. They are favored over less optimal designs with concentrations that lie between *c*_tol_ and *c*_tox_.

#### Choice of cost function parameters

The cost function described above provides a set of calibration parameters that offer flexibility in balancing the relative importance of efficacy vs. toxicity. While the two concentration thresholds *c*_eff_ and *c*_tox_ need to be determined experimentally, the remaining four parameters *c*_tol_, *γ*_thr_, *ϑ*_thr_ and *ϑ*_lim_ allow calibration of the stringency of the toxicity constraint. Thus, it might be deemed acceptable to live with a certain level of wall toxicity in some cases but not in others, and these adjustable parameters allow this form of fine tuning. In light of the severe consequences of delayed endothelial healing [42, 43] and in view of currently available experimental data, we chose the calibration parameters summarized in Table 2.

**Table 2:** Calibration parameters of the cost function.

Drug	*c*_tol_	*γ*_thr_	*ϑ*_thr_	*ϑ*_lim_	*c*_eff_ (mol m^−3^)	*c*_tox_ (mol m^−3^)
Paclitaxel	90% ⋅ *c*_tox_	1%	1%	5%	1 × 10^−5^	1 × 10^−2^
Sirolimus	90% ⋅ *c*_tox_	1%	1%	5%	1 × 10^−5^	0.73

The choice of parameter values for *c*_tol_, *γ*_thr_, *ϑ*_thr_ and *ϑ*_lim_ is rather conservative and is expected to allow us to avoid arterial wall toxicity. Lacking experimental data on the relative severity of toxic concentration in the different layers of the arterial wall covered by each toxicity score, we (arbitrarily) assume the three toxicity scores to be of equal importance. As a consequence, we choose the same set of calibration parameters for each of the toxicity scores and a weight factor of $\frac{1}{3}$ multiplying each toxicity score of the cost function. It should be recognized, however, that new experimental results or clinical studies might lead to future changes in some or all of these parameters as well as choosing a different set of parameters for each individual score.

Both paclitaxel and sirolimus inhibit the proliferation of SMCs and ECs by arresting the cells at a point in their cell cycle. At sufficiently high concentrations, paclitaxel is cytotoxic and leads to cell death [7]. Values for toxic paclitaxel concentrations are available in the literature [23, 44]. The values of minimum efficacious concentration and toxic concentration for paclitaxel used in the present work, *c*_eff_ = 1 × 10^−5^ mol m^−3^ and *c*_tox_ = 1 × 10^−2^ mol m^−3^, are in agreement with [45]. These values are based on the work of [46] who studied the inhibitory effect of paclitaxel on cancer cells. This value of the minimum efficacious concentration is in good agreement with earlier work of [47], who reported prolonged inhibition of human arterial SMCs growth at a medium concentration of 1 × 10^−4^ mol m^−3^ with an IC_50_ value for paclitaxel on human arterial SMC growth of 2 × 10^−6^ mol m^−3^.

Unlike paclitaxel, sirolimus is a cytostatic agent, i.e. its arrest of the cell cycle is not associated with cell death even at a relatively elevated concentration [7]. This behavior allows for a wider therapeutic window for sirolimus than for paclitaxel. [48–50]) all report IC_50_ ≈ 1 × 10^−6^ mol m^−3^ for human SMC proliferation and/or migration. This is of the same order of magnitude as the IC_50_ value for paclitaxel for human arterial SMC growth. We thus set the value of the minimum efficacious concentration for sirolimus to *c*_eff_ = 1 × 10^−5^ mol m^−3^, which is the same value as that for paclitaxel. The toxic threshold of sirolimus is less clear, and only limited toxic reactions to sirolimus have been reported [51, 52]. We thus set the toxic concentration threshold to 200% of the maximum binding site density of sirolimus. These threshold values for the baseline case are summarized in Table 2. In order to study the effect of these threshold choices on our results, we will also present results for a case where these thresholds were multiplied or divided by a factor of 10. The effect of total drug dose (integral of drug concentration over time) is not considered as a separate metric in the present cost function because the experimental evidence in the literature of the effect of drug dose is too sparse to be translated into a reliable metric.

#### Practical considerations

To avoid spending valuable calculation time on undesirable parameter configurations, we implemented additional criteria terminating the evaluation process of the cost function if the toxicity parameter *ϑ*_m_ surpasses a value of 5% or if the maximum total concentration in the media *c*_T,m_ drops below the efficacious limit at any time after day 5 of simulated time. The latter criterion automatically discards designs that do not lead to efficacious concentration levels within that five day period. If the simulation is terminated early, we take the score calculated at the last computed time point prior to termination as the basis value for the remaining time points in the averaging process. The toxicity parameter was capped at a maximum value *T*_max_ = 10, since this already corresponds to a ten-fold increase over what by design would be considered an undesirable situation.

To impose a gradient in the case of non-efficacious designs, we add the quantity $\left[1-\frac{\text{max}(c_{\text{T,m}})}{c_{\text{eff}}}\right]$ to ${\bar{I}}_{\text{m}}$. Based on typical DES release times and motivated by the pathobiology following stent implantation, we set four weeks as the target simulation time *t*_end_ [53–56]. A sensitivity study of the cost function has shown that our results are fairly insensitive to a variation of the evaluation period between one and eight weeks.

To keep the simulation time of each function evaluation to a minimum and as has already been mentioned, we only include three stent struts in our numerical model. Thus, the overall stent length simulated is considerably smaller than real stents. Our simulations have shown that, with the exception of the first and last strut, the concentration distribution around the struts within the arterial wall is symmetric and almost identical around the central struts [11]. Since this concentration distribution serves as the basis for the evaluation of our cost function, we can approximate a longer stent by appropriately multiplying the score obtained for the symmetric mid-section of our strut setup. A sensitivity analysis has revealed that the final results are only minimally sensitive to this form of stent elongation.

#### Optimization framework

We use a SMF-type optimization algorithm [57] to minimize the cost function *J*_ETB_. Here we will only outline the basic concepts of this method. The reader is referred to [31] for more details.

The SMF method belongs to the class of pattern search algorithms for numerical optimization. These algorithms minimize a given cost function by gradually exploring the space of possible designs. In our case, each combination of drug concentration initially loaded onto the stent *c*_0_ and drug release time *t*_E_ constitutes a point in the design space.

Booker *et al.* (1999) [57] introduced the idea of a global *search step* that efficiently leverages information obtained from previously evaluated points of the design space. All previously evaluated points are used to create an approximation of the cost function hypersurface which serves as an inexpensive surrogate to scan for and identify potential new minimum points in the entire design space. As long as the potential minimum points revealed by the search step yield improved cost function values, the information from these points is added to the approximation surface and a new search is performed.

**Fig 3:**
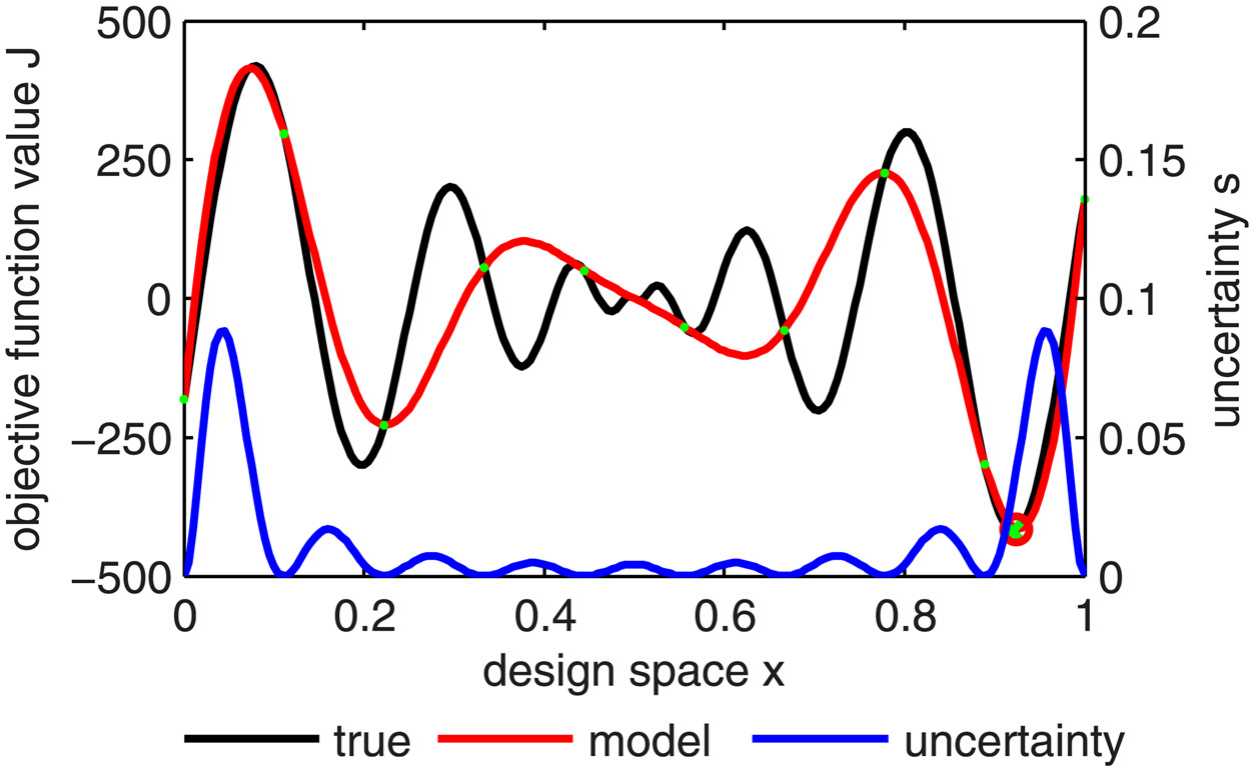
One-dimensional example of a Kriging interpolation hypersurface (red) of the true cost function hypersurface (black) based on 10 evaluated points *P* (green) with uncertainty *s* (blue) over the design space *x*. The design space *x* and the cost function values form the cost function hyper-space. The true cost function minimum (green cross) could be almost perfectly identified by the Kriging model (predicted minimum marked by red circle) with only 10 points.

The most common interpolation method used in this context is the Kriging method [58, 59]. A major advantage of this interpolation (and extrapolation) method is that on top of an estimation of the function value, it also provides the level of uncertainty in the estimated value (Fig 3). This information can then be used to enhance the search procedure: instead of minimizing the Kriging interpolant directly, new prospective optimum points are identified by maximizing the probability of improvement over the current optimum by a predefined margin [20].

**Fig 4:**
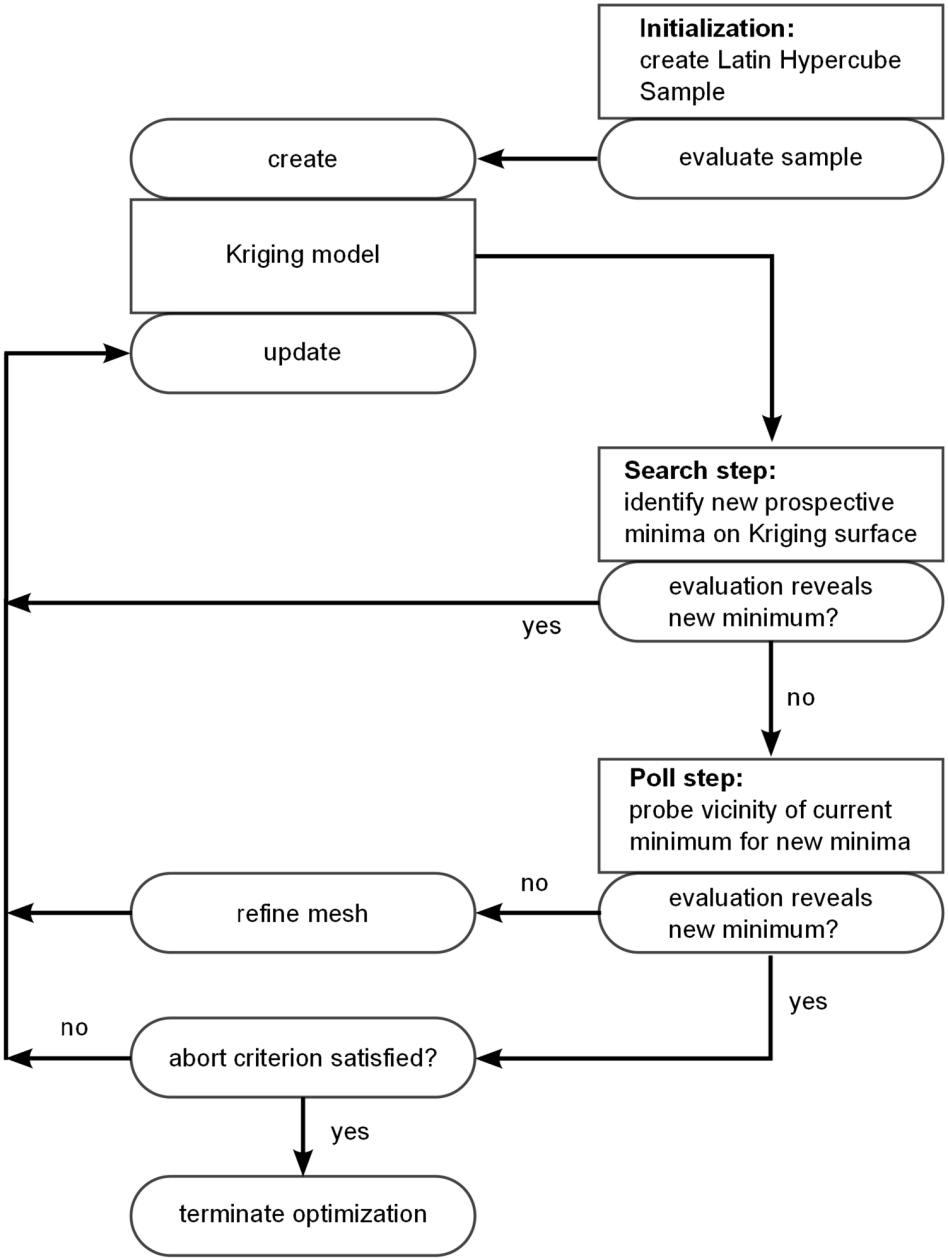
Flow diagram of the optimization algorithm and its coupling to the computational model.

The optimization procedure (Fig 4) begins with the evaluation of a set of initial sampling points to create the very first surrogate surface. We use the latin hypercube sampling algorithm presented in [60] to ensure a uniform distribution of these points throughout the design space. We use 20 initial designs for our 2-dimensional design space.

When a search step fails to yield a new minimum, a *poll step* is initiated. Starting from the current minimum point of the design space, a set of new points is chosen in the design space. The choice of these points is restricted to a grid which discretizes the design space and defines the search pattern. The cost function values at these points are then evaluated (a process referred to as *polling*). If any one of these points yields a cost function value that is lower than the value associated with the initial point, then this point becomes the new minimum point. If, on the other hand, none of these points is able to improve the cost function, then the grid spacing is refined and a new set of points is chosen on this new grid. A new search step succeeds the poll step, independent of its outcome, taking into account all newly evaluated designs and their respective computed value of the cost function. We consider the design space to have been fully explored once the mesh of the poll step is refined 10-fold, all points neighboring the current minimum point have been polled, and the search step fails to identify new optimum points in five consecutive search steps. At this point the optimization is terminated.

**Fig 5:**
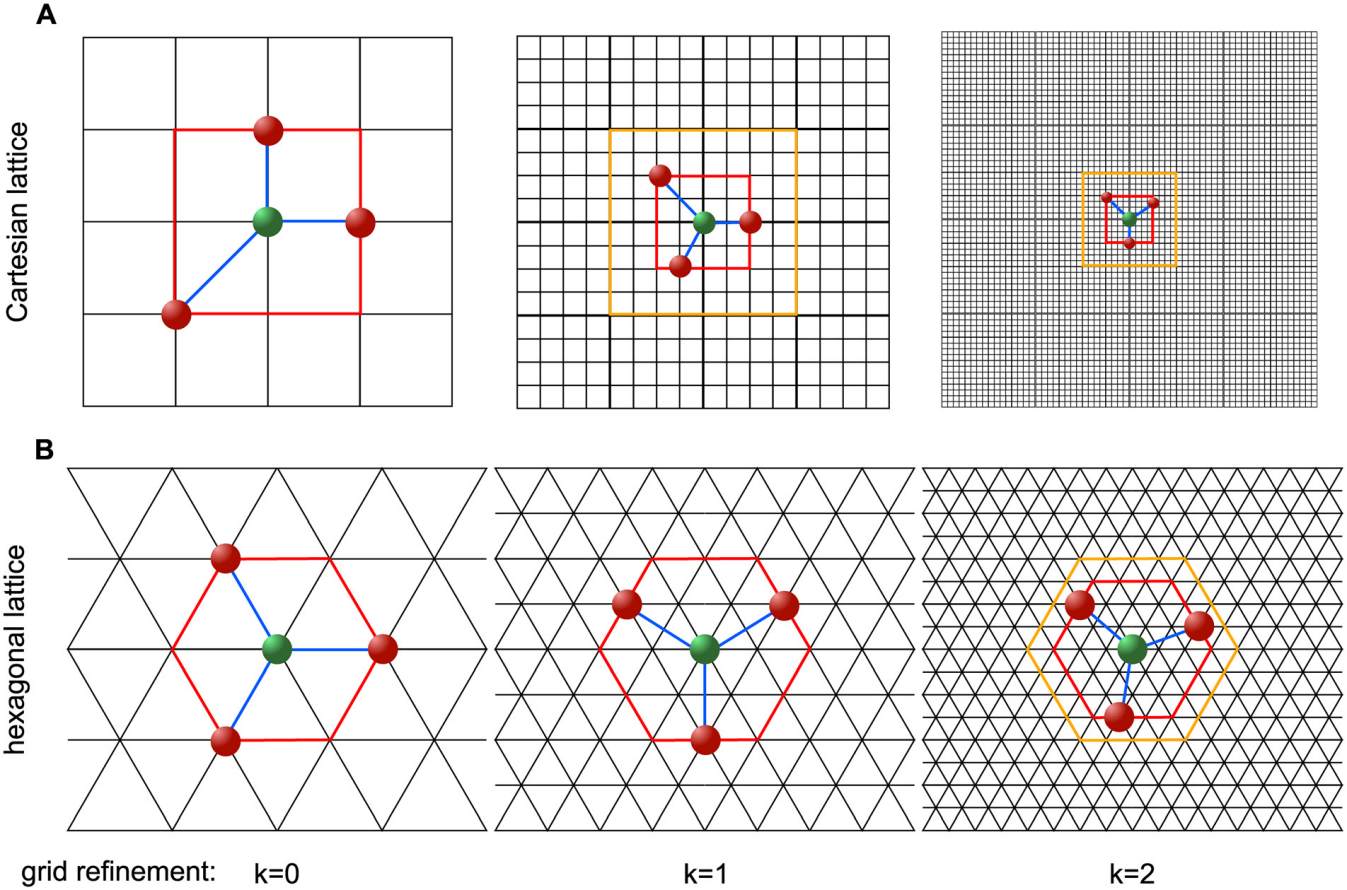
Polling the design space. **A**: Two consecutive factor of 4 grid refinements and factor of 2 shell of prospective polling points refinements of the LT-MADS algorithm on a 2-dimensional Cartesian lattice; **B**: Two consecutive factor of 2 mesh refinements of the *λ*-MADS algorithm on a hexagonal lattice *A*_2_ with a shell of prospective polling points at a distance of 1, 2 and 3 grid points for the initial grid (*k* = 0) and after *k* = 1 and *k* = 2 consecutive grid refinements, respectively. Search directions (in blue) of a minimal positive basis connect the current optimum point (in green) with the selected poll designs (red). Current shell of prospective polling points is marked in red, previous shell of prospective polling points is marked in orange.

The following two key novelties have been implemented in the present SMF algorithm leading to improved performance of the optimization procedure: 1) The arrangement of different designs in the design space is on a grid with a higher number of directly neighboring designs than in the case of the typically used Cartesian grid [14, 61–64]. Search and poll steps are always performed on a “laminated” lattice [31], which maximizes the regularity and density of the grid points for the respective dimension of the design space. 2) The factor by which the grid is refined after a failed poll step is reduced compared to other algorithms so that the number of designs that can be investigated in each poll step increases without having to pick designs that are very close to the current optimal point (see Fig 5). This new mesh adaptive direct search (MADS) algorithm is dubbed “*λ*-MADS” [31].

### Optimization cases and performance

We will present a total of 12 different optimization runs. To generate each detailed map of the cost function as presented in the following section requires approximately 10 days using a total of 8 cores of an Intel Xeon CPU X5680 @ 3.33GHz processor. However, the algorithm permits identification of the optimal region after 3 days. The additional week is required to refine the initial cost function map.

The optimization results are divided into two groups: the first group addresses the optimization of the drug release strategy for paclitaxel and sirolimus assuming the baseline values for the efficacy and toxicity threshold and investigates the effect of varying these thresholds on the optimization results; the second group investigates the effect of an increasing SMC fraction in the SES on the optimal drug release strategy for paclitaxel and sirolimus.

## Results

We wish to determine the optimal drug delivery strategy, defined here as the combination of the drug concentration initially loaded onto the stent *c*_0_ and the drug release time *t*_E_ as characterized by the diffusion coefficient of the drug *D*_c_ within the 10 μm-thick polymeric matrix in which it is embedded. The optimization is performed for both paclitaxel and sirolimus.

### Physical insight into the effect of release kinetics on arterial wall drug concentration

**Fig 6:**
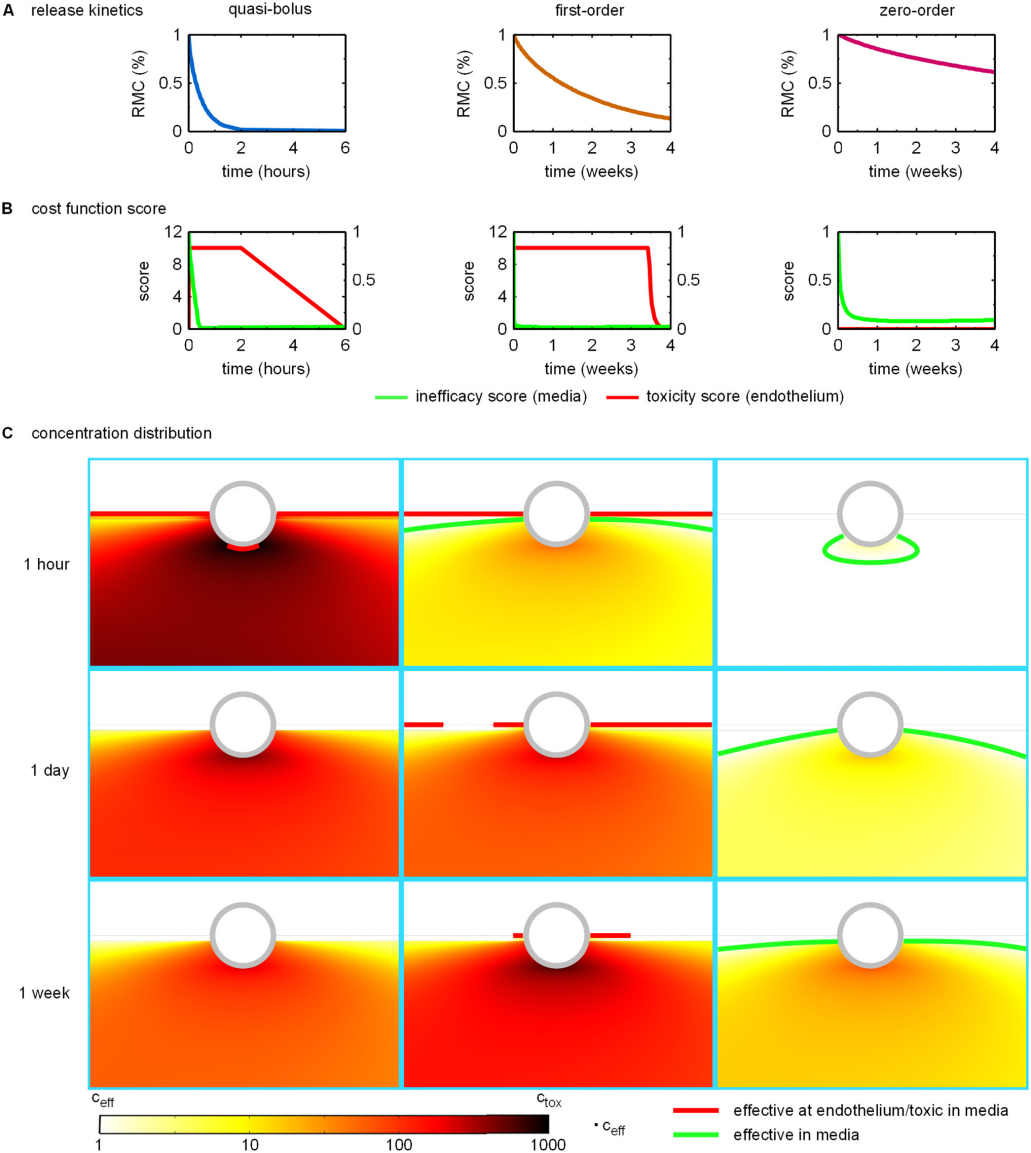
Release kinetics and resulting cost function scores and normalized concentration distributions for repesentative designs of *quasi-bolus*, *first-order*, and *zero-order* drug release. **A**: Release profiles as quantified by the time evolution of the remaining mass percentage of drug in the polymer coating (RMC). **B** Time evolution of the endothelial toxicity score ${\bar{T}}_{\text{e}}$ (left y-axis) and the inefficacy score ${\bar{I}}_{\text{m}}$ (right y-axis). **C**: Contour plots of the concentration distribution normalized by the minimum efficacious concentration *c*_eff_ at 1 hour (*first row*), 1 day (*second row*) and 1 week (*third row*) post stent implantation. The highest concentration values encountered close to the stent strut surface are colored in black indicating toxic concentrations. A green contour line marks the threshold between efficacious and non-efficacious drug concentration in the media. If the contour is missing, the entire depicted domain is exposed to efficiacious concentrations. The red countour lines enclose toxic concentration regions in the media and mark concentration levels at the endothelial surface that are unacceptably elevated.

Before describing the optimization results, we will focus on three concrete examples of paclitaxel release strategies and their resulting drug concentration distributions (Fig 6). This allows us to gain some physical insight into the optimization results.

Fig 6A shows the time evolution of the percentage of the remaining drug mass in the polymer coating resulting from three different release kinetics. The first column depicts release kinetics where all of the drug contained in the polymer is released within the first few hours following DES implantation, i.e. in a quasi-bolus fashion. The stent emptying time scale *t*_E_ is significantly shorter than either the time period considered in our simulations (4 weeks) or the largest time scale in the transport problem, namely the drug unbinding time. In the second column, drug release occurs over a period of one month, which is comparable to the drug unbinding time scale so that drug release rate becomes concentration-dependent. We will denote these release kinetics as first-order. In the third column, the drug is released so slowly that most of the drug is not released during the 4-week period considered. Drug release occurs at a quasi-steady rate with a stent emptying time scale (*t*_E_ = 5 months) significantly longer than all other time scales involved in drug transport. We will call this type of release kinetics zero-order or long-term release.

Fig 6B depicts the time evolution of the endothelial toxicity score and the inefficacy score resulting from the three different release strategies outlined above: *t*_E_ = 1 h, *c*_0_ = 2.5 mol m^−3^ (quasi-bolus release), *t*_E_ = 1 month, *c*_0_ = 10 mol m^−3^ (first-order release) and *t*_E_ = 5 months, *c*_0_ = 3 mol m^−3^ (zero-order release). For the quasi-bolus release, we can see that almost immediately following the beginning of drug release, the toxicity score spikes to the maximum allowed value of 10 while within the first half hour the media gets flooded with drug at a concentration leading to efficacious concentration levels throughout the therapeutic domain (very small inefficacy score values). This maximum toxicity score is maintained for the the first two hours within which the polymer is almost entirely depleted of drug. The toxicity score then gradually drops to zero within the following 4 hours. The inefficacy score stays at ≈ 0.01 throughout the hours following polymer depletion before eventually increasing as drug is gradually washed out of the media (not shown in Fig 6B). By the end of the 4-week period (data not shown), the inefficacy score rises to ≈ 0.16 (data not shown).

Similar to the quasi-bolus release, the endothelial toxicity score for the first-order release immediately spikes to the maximum allowed value. However, contrary to the quasi-bolus case, the endothelial toxicity score remains at the maximum value until 3.5 weeks after stent implantation and only then begins to rapidly decrease to a value of zero at the 4-week mark. The inefficacy score drops to less than 0.2 within the first hour of release, further decreasing over the next few hours to ≈ 0.02 where it remains for the duration of the 4-week period.

In the case of the zero-order release, the endothelial toxicity score is zero throughout the considered period, while the inefficacy score drops to ≈ 0.25 within the first day of drug release and attains ≈ 0.1 at the 4.5-day mark. The score drops to a minimum of ≈ 0.08 within 10 days and then stays around this value for the remainder of the 4-week period.

Fig 6C depicts the concentration distributions (normalized by the efficacious concentration threshold *c*_eff_) around the stent strut furthest downstream (third strut) at the 1-hour, 1-day, and 1-week marks for the design points representative of quasi-bolus release, first-order release, and zero-order release. The quasi-bolus drug release (left column in Fig 6C) leads to the release of all of the drug within the first few hours after implantation. Accordingly, the highest drug concentrations are attained within the first hour with local concentrations in the media near the stent strut exceeding the toxicity limit and unacceptably high concentrations in the SES (as indicated by the red contour line at the luminal surface). At the 1-hour time point, drug concentrations are at efficacious levels over practically the entire media. The next two time points illustrate how the drug concentration decays over the following week. After only one day, the concentrations at the luminal surface are sufficiently low to allow EC proliferation and migration. Because the characteristic time for drug release in this case is considerably shorter than all of the time scales characterizing drug transport and reaction (convection, diffusion, as well as drug binding and unbinding), the quasi-bolus release strategy can be thought of as a transport-limited case.

For the first-order drug release kinetics (middle column), large parts of the media are already exposed to efficacious concentration levels at the 1-hour time point (indicated by the green contour line), whereas the SES experiences drug concentrations that are sufficiently elevated to inhibit EC wound healing (red contour line). After a day, concentration levels in the media continue to increase, and drug concentration throughout most of the SES remains beyond the acceptable level for endothelial wound healing. At the 1-week mark, concentration levels in the media close to the strut have nearly attained their maximum concentration level of more than 600 times the efficacious concentration threshold. At the luminal surface, drug concentration levels have dropped but are still sufficiently high to inhibit EC proliferation and migration.

For the zero-order drug release kinetics (right column), the concentrations within the first hour attain the efficacious threshold in the media only in the vicinity of the stent struts. After one day, the concentration levels in most of the media are efficacious, except for the zones in between struts close to the luminal surface. The steady release over the next week slowly increases concentration levels throughout the media leaving almost no zones of the media exposed to non-efficacious concentrations. The highest concentrations observed in the media close to the struts at the 1-week mark are around 70 times the efficacious concentration threshold. Concentration levels in the SES or at the luminal surface are never sufficiently high to impair EC wound healing. Since all time scales of the transport and reaction problem in the arterial wall are significantly shorter than the release time scale in this case, this scenario can be viewed as a quasi-steady state for transport in the arterial wall and hence a release-limited situation.

### Baseline optimization results and their sensitivity to the concentration thresholds

**Fig 7:**
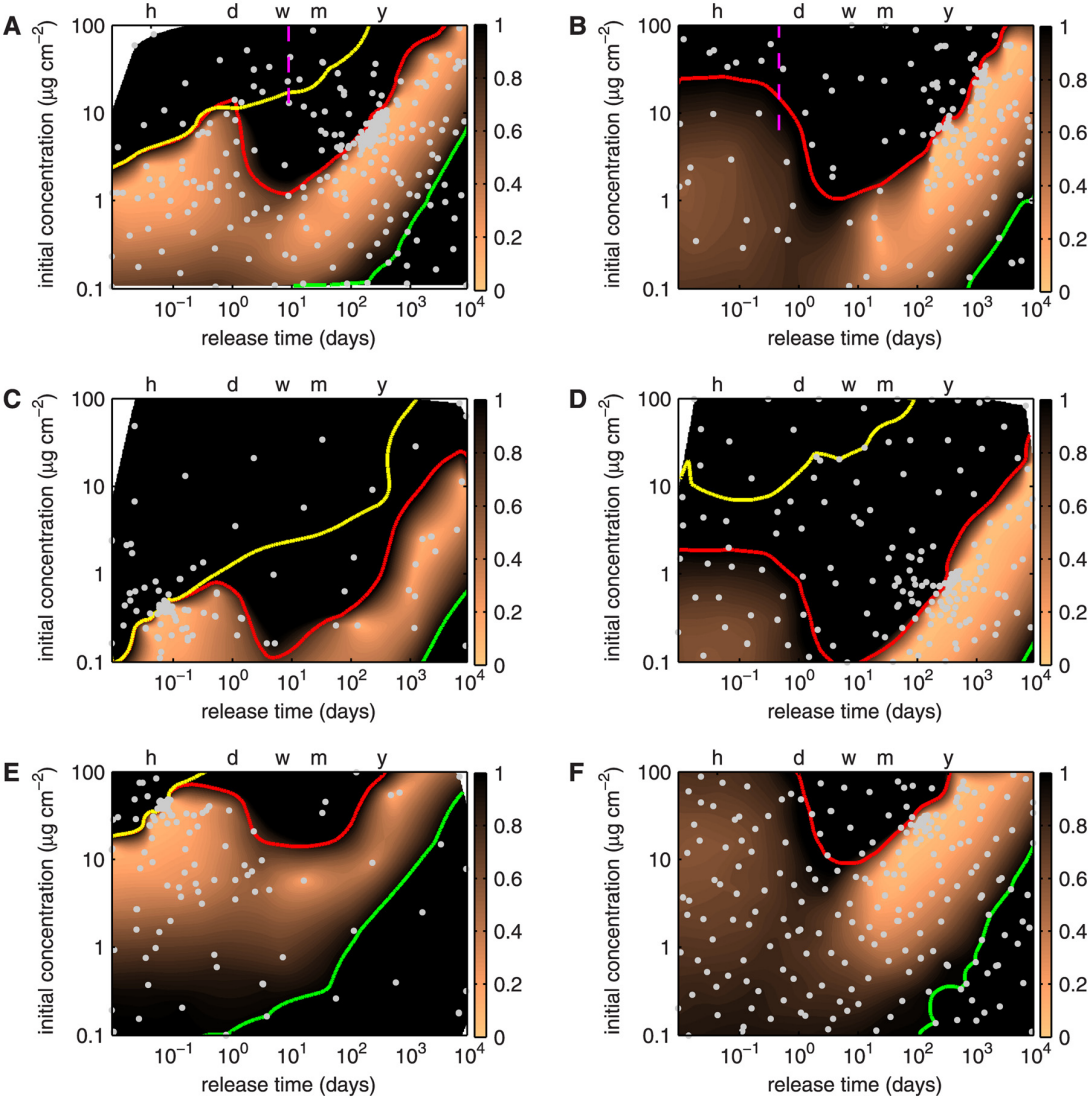
Contour plots of the cost function for paclitaxel (left column) and sirolimus (right column) over the design space consisting of *initial concentration in the stent polymer* *c*_0_ and *release time* *t*_E_. The scale for the cost function representation is truncated at a maximum of 1; all values larger than 1 are colored black. The dashed magenta lines in panels A and B mark the time scales for drug unbinding. The green contour line traces ${\bar{I}}_{\text{m}}=1$, the yellow contour line ${\bar{T}}_{\text{m}}=1$, and the red contour line ${\bar{T}}_{\text{e}}=1$. The horizontal axis at the top of the plot marks the time points of 1 (h)our, 1 (d)ay, 1 (w)eek, 1 (m)onth and 1 (y)ear. Gray dots indicate evaluated designs. Optimization cases **A**: paclitaxel release and **B**: sirolimus release with baseline cocnentration thresholds. **C**: Paclitaxel release and **D**: sirolimus release with concentration thresholds reduced by a factor of 10. **E**: Paclitaxel release and **F**: sirolimus release with concentration thresholds increased by a factor of 10.

Fig 7A shows the surface of the cost function obtained using the baseline model setup for paclitaxel elution. The plot is the result of a natural neighbor interpolation [65] of the evaluated designs indicated by the gray dots. In analogy to topographical maps (equating function value magnitude with elevation), we can describe the the cost function as a mountain range with two valleys that define two optimal regions. We will refer to the directions of increasing concentrations and release time as north and east, respectively. Three colored contours are shown in Fig 7A: the green contour line traces ${\bar{I}}_{\text{m}}=1$ and thus defines the border for inefficacious designs; the yellow contour line traces ${\bar{T}}_{\text{m}}=1$ and thus marks the threshold of toxicity in the media; the red contour line traces ${\bar{T}}_{\text{e}}=1$ and hence demarcates concentrations above which inhibition of EC wound healing would be expected to occur. It should be noted that the interpolated surface is only as good as the underlying evaluated designs (indicated by gray dots). Even if this fact limits our ability to reach detailed conclusions in some local regions of the design space, the overall conclusions are unaffected.

The optimization results reveal two optimal regions. The first lies in a relatively flat valley spanning an initial concentration range of 0.6 to 2.5 μg cm^−2^ and covering a release time of a few minutes to 4 hours. The cost function score ranges between 0.08 and 0.15 in this valley. The rapid drug release of these designs causes toxic concentrations in the media and unacceptably high drug concentrations at the EC surface (${\bar{T}}_{\text{m}}<0.01$ and ${\bar{T}}_{\text{e}}<0.06$) over very short periods of time. The second optimal region is in a chasm beginning at a release time scale of ≈ 1.5 months and an initial concentration of ≈ 1 μg cm^−2^ and ending at a release time of ≈ 12.5 years and an initial concentration of ≈ 40 μg cm^−2^. Designs in this chasm have a cost function score of less than 0.1. The most optimal designs with a cost function of less than 0.05 lie in a crevice bounded by the designs *t*_E_ ≈ 5.5 months, *c*_0_ ≈ 4 mol m^−3^ and *t*_E_ ≈ 1 year, *c*_0_ ≈ 9 mol m^−3^. These designs do not lead to any toxicity. Designs in this second region with a cost function score of less than 1 are bound on the northwestern end by the region of unacceptably high drug toxicity at the luminal surface (delineated by the red contour line) and on the southeastern side by the zone of unacceptably low drug efficacy in the media (green contour line). It is interesting to note that designs corresponding to today’s paclitaxel-eluting stents (P-DES) release the drug over a period of a few weeks to a few months with initial concentration loads above 10 μg cm^−2^ [66] and thus lie on top of the central mountain in the north of our landscape.

Fig 7B depicts the baseline optimization results for sirolimus elution. The results offer a very different picture from the case of paclitaxel: there is only a single band of optimal release (*J* < 0.05) starting at a characteristic release time of ≈ 5 months with an initial concentration of ≈ 1.4 μg cm^−2^ and ending at a release time of ≈ 17.5 years and an initial concentration of ≈ 56 μg cm^−2^. Designs in this chasm do not lead to any toxicity. Similar to the case of paclitaxel, the band of designs with a cost function score of less than 1 are bounded by the region of unacceptably high drug toxicity at the luminal surface (red contour line) and the zone of unacceptably low drug efficacy in the media (green contour line). A yellow contour line does not appear in this figure because even the highest concentrations considered remain below the medial toxicity levels. Similar to the case of paclitaxel, today’s sirolimus-eluting stents (S-DES) release the drug over a period of weeks to a few months with initial concentration loads above 10 μg cm^−2^ [67, 68] and thus lie on top of the central mountain in the north of the landscape.

The results in Fig 7A and 7B were obtained using the baseline values of threshold concentrations for drug efficacy and toxicity. Fig 7C–7F depict the sensitivity of the optimization results to these threshold values. Fig 7C reveals that decreasing the thresholds for the efficacious and toxic concentration limits for paclitaxel by a factor of 10 simply shifts the contour lines southward to about 10-fold lower initial concentrations. The principal features that we identified for the baseline conditions (Fig 7A) remain unchanged: two optimal regions, one in the very fast release region (release time of a few minutes to ≈ 4 h with initial drug concentrations ranging from ≈ 0.1 μg cm^−2^ to ≈ 0.35 μg cm^−2^) and one in the very slow release chasm (with the most optimal designs bounded by the two designs *t*_E_ ≈ 4 months, *c*_0_ ≈ 0.2 mol m^−3^ and *t*_E_ ≈ 4.5 years, *c*_0_ ≈ 2.2 mol m^−3^). Contrary to the baseline case (Fig 7A), the fast release strategy is slightly more favorable than the slow release strategy, as demonstrated by the high density of evaluation points (gray dots) in this region.

Fig 7D shows that a 10-fold decrease in the thresholds for the efficacious and toxic concentrations has a similar effect on the optimization of sirolimus release as it does on paclitaxel release. The principal features of the cost function map remain largely the same with the optimal stent loading concentrations reduced by approximately an order of magnitude. The most optimal release strategies (*J* < 0.05) are found in the slow release valley bounded by the two designs *t*_E_ ≈ 8 months, *c*_0_ ≈ 0.4 mol m^−3^ and *t*_E_ ≈ 1.5 years, *c*_0_ ≈ 1 mol m^−3^. The reduced toxicity threshold in the media leads to the appearance of the yellow contour line that defines a medial toxicity of ${\bar{T}}_{\text{e}}=1$.

Increasing the thresholds for the efficacious and toxic concentrations leads to a shift northward of the optimal cost function values towards higher initial concentrations. For the release of paclitaxel (Fig 7E), the most optimal release strategies lie in the fast release valley characterized by release times of a few minutes to ≈ 4 h at initial concentrations ranging from ≈ 10 μg cm^−2^ to ≈ 35 μg cm^−2^). Optimal sirolimus release strategies lie in the crevice formed by the two designs *t*_E_ ≈ 3 months, *c*_0_ ≈ 20 mol m^−3^ and *t*_E_ ≈ 7 months, *c*_0_ ≈ 35 mol m^−3^ (Fig 7F).

### Sensitivity of the optimization results to smooth muscle cell content in the subendothelial space

**Fig 8:**
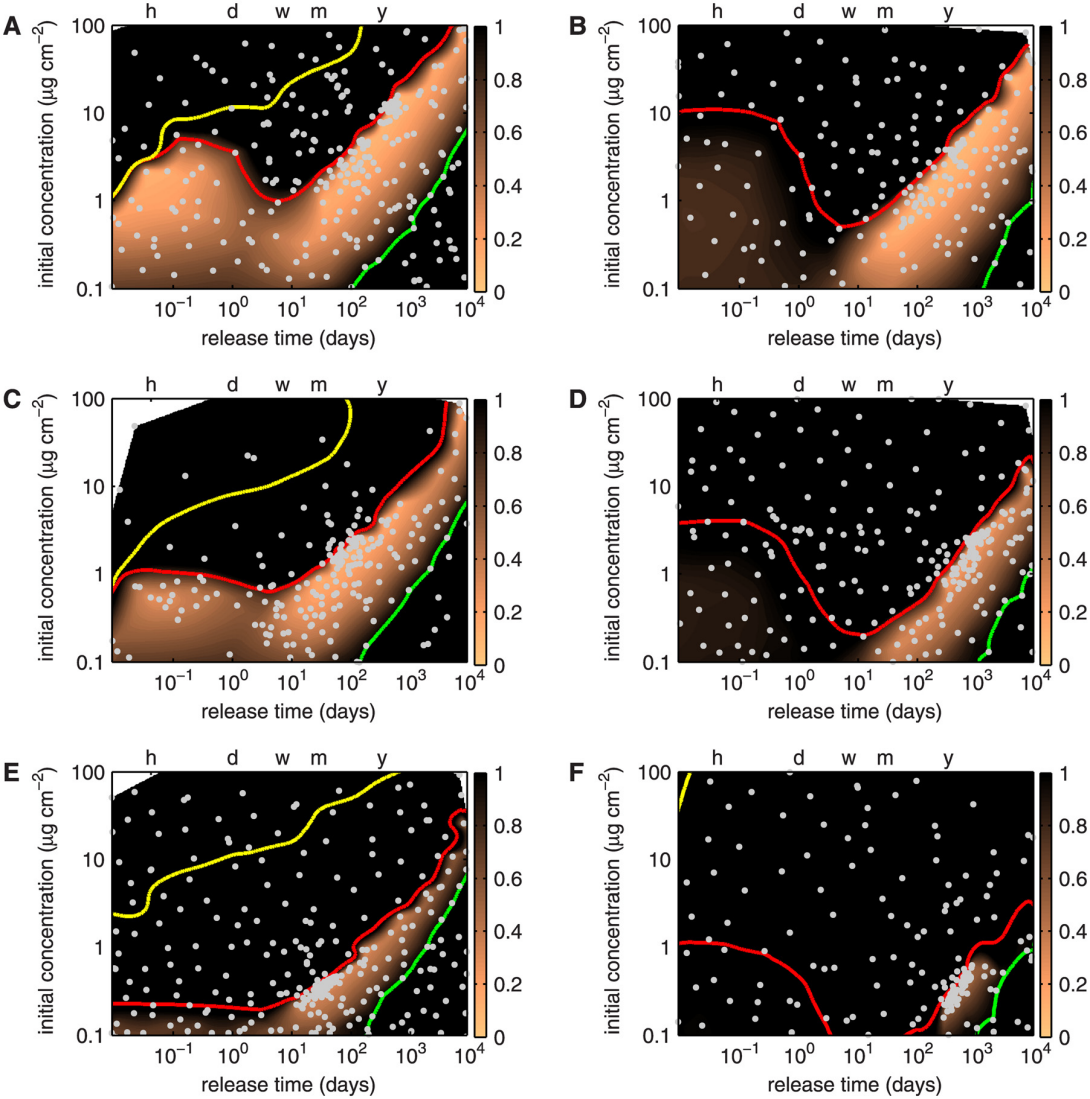
Contour plots of the effect of the presence of SMCs in the SES on the cost function for paclitaxel (left column) and sirolimus (right column) over the design space consisting of *initial concentration in the stent polymer* *c*_0_ and *release time* *t*_E_. The scale for the cost function representation is truncated at a maximum of 1; all values larger than 1 are colored black. The green contour line traces ${\bar{I}}_{\text{m}}=1$, the yellow contour line ${\bar{T}}_{\text{m}}=1$, and the red contour line ${\bar{T}}_{\text{e}}=1$. The horizontal axis at the top of the plot marks the time points of 1 (h)our, 1 (d)ay, 1 (w)eek, 1 (m)onth and 1 (y)ear. Gray dots indicate evaluated designs. Optimization cases **A**: paclitaxel release and **B**: sirolimus release with SES SMC density corresponding to 1% of the medial SMC density. **C**: Paclitaxel release and **D**: sirolimus release with SES SMC density corresponding to 5% of the medial SMC density. **E**: Paclitaxel release and **F**: sirolimus release with SES SMC density corresponding to 25% of the medial SMC density.

Unlike healthy arteries, atherosclerotic vessels contain SMCs in the SES. The presence of SMCs in the SES alters drug transport within this layer and necessitates the incorporation of drug reaction with the SMCs in the SES. Fig 8 depicts the optimization results for paclitaxel and sirolimus for progressively increasing fractions of SMCs in the SES, expressed as a percentage of the SMC volume fraction in the media.

Fig 8A demonstrates that when the SES SMC density is 1% of the medial SMC density, two optimal zones, a very fast release optimum around *t*_E_ ≈ 1 h, *c*_0_ ≈ 1.9 mol m^−3^ with a cost function score of *J* ≈ 0.15 and a slow release optimum in a chasm bound by *t*_E_ ≈ 5 months, *c*_0_ ≈ 3.7 mol m^−3^ and *t*_E_ ≈ 1.5 years, *c*_0_ ≈ 12.5 mol m^−3^ with a cost function score *J* ≈ 0.1 exist. The medial toxicity and inefficacy contour lines are basically unaltered compared to the baseline case (cf: Fig 7A). The endothelial toxicity contour moves slightly southward. To quantify this shift, we report in Table 3 the approximate *c*_0_ on this contour at the four time points *t*_E_ = 1 h, *t*_E_ = 1 week (corresponding to the tip of the ridge separating the two optimum zones), *t*_E_ = 1 month, and *t*_E_ = 1 year.

**Table 3:** Approximate initial concentration (in mol m^−3^) at distinct time points on the endothelial toxicity contour line for paclitaxel and sirolimus release strategy optimization.

Paclitaxel	*t*_E_ = 1 h	*t*_E_ = 1 week	*t*_E_ = 1 month	*t*_E_ = 1 year
Baseline	3.5	1	1.9	10.6
1% medial SMC	2.7	0.9	1.85	10
5% medial SMC	1	0.6	1	6.25
25% medial SMC	0.2	0.2	0.35	1.9
Sirolimus	*t*_E_ = 1 h	*t*_E_ = 5 days	*t*_E_ = 1 month	*t*_E_ = 1 year
Baseline	27	1	1.6	8.9
1% medial SMC	11	0.5	0.85	5.7
5% medial SMC	4	0.24	0.28	1.3
25% medial SMC	1.1	0.06	0.1	0.33

Fig 8B depicts the results of the sirolimus release optimization assuming an SES SMC density of 1% of the medial SMC density. Similar to the baseline case (cf: Fig 7B), we can identify a single slow release optimum in a chasm bound by *t*_E_ ≈ 3 months, *c*_0_ ≈ 0.9 mol m^−3^ and *t*_E_ ≈ 4.5 years, *c*_0_ ≈ 11 mol m^−3^ with a cost function score *J* ≈ 0.1. The medial inefficacy contour line is basically unaltered compared to the baseline case, whereas the endothelial toxicity contour shifts slightly southward (quantified in Table 3).

When the SES SMC content is increased to 5% of the medial SMC density, the fast release optimum zone for paclitaxel is reduced more drastically than the slow release optimum zone (Fig 8C). The optimal fast release strategy has a release time of approximately *t*_E_ = 1 h at an initial concentration of *c*_0_ ≈ 0.5 mol m^−3^ yielding a cost function score of *J* ≈ 0.3. The optimal slow release crevice is bound by the two designs *t*_E_ ≈ 2 months, *c*_0_ ≈ 1 mol m^−3^ and *t*_E_ ≈ 5 months, *c*_0_ ≈ 2.3 mol m^−3^ with a cost function score *J* ≈ 0.15. In the case of sirolimus, the optimal slow release zone shrinks significantly (Fig 8D). An optimal release strategy with a cost function score of *J* ≈ 0.2 can only be found at *t*_E_ ≈ 2 years and an initial concentration *c*_0_ ≈ 2 mol m^−3^. The endothelial toxicity contour moves significantly southward for both paclitaxel and sirolimus as quantified in Table 3.

Increasing the SES SMC content further to 25% of the medial SMC density leaves only a very narrow area between the endothelial toxicity and medial inefficacy contour lines for both drugs (Fig 8E and 8F). Optimal designs for paclitaxel under these conditions can be found along a thin line bounded by *t*_E_ ≈ 14 days, *c*_0_ ≈ 0.19 mol m^−3^ and *t*_E_ ≈ 2 years, *c*_0_ ≈ 2 mol m^−3^, yielding a cost function score of *J* ≈ 0.3. The approximate “initial concentration locations” on the endothelial toxicity contour are reported in Table 3. When the SES SMC content is increased to 25% of the medial SMC density, the optimal sirolimus release strategy only yields a cost function score of *J* ≈ 0.5 with *t*_E_ ≈ 1 year, *c*_0_ ≈ 0.3 mol m^−3^ (Fig 8F). The endothelial toxicity contour moves further southward for both drugs (Table 3).

## Discussion

In this paper, we used the Surrogate Management Framework to optimize drug delivery from P-DES and S-DES. The objectives of the optimization were to obtain drug concentrations in the media that are efficacious against restenosis but yet are subtoxic, while simultaneously targeting drug concentrations at the EC surface that are sufficiently low so as not to inhibit endothelial wound healing. The two design parameters in the optimization were the drug release rate from the stent and the initial drug concentration loaded onto the stent. The results revealed dramatically different optimal release strategies for the two drugs, primarily attributable to differences in the kinetics of their reaction. We now wish to summarize our findings and to gain insight into the physical basis of the optimization results. Given the assumptions that we have made and the degree of simplification in our numerical model of drug transport, we will refrain from denoting one particular combination of design parameters as *the* optimum and rather focus on a broader description of regions in the design space identified in the optimization.

### Paclitaxel-eluting stents either require quasi-bolus or zero-order drug release kinetics to avoid adverse concentration levels at the endothelium

Optimization of P-DES led to a design space that is divided into four zones. The first zone is characterized by initial drug concentrations higher than 10μg cm^−2^. This zone has no acceptable designs except for drug release kinetics with a drug release time longer than one year. The sub-10μg cm^−2^ area contains the remaining three zones of which only designs with either quasi-bolus or zero-order release kinetics lead to acceptable outcomes, while any designs with first-order release kinetics result in undesirable conditions in the arterial wall that are far from optimal. This conclusion holds regardless of the actual magnitude of the efficacy and toxicity thresholds.

The simulation results demonstrate that what primarily limits the efficacy of a particular P-DES design is the excessive supply of drug to the SES, leading to concentrations that are expected to inhibit EC proliferation and migration. In the baseline computations, we assumed that the SES was free of SMCs so that drug concentration in the SES is determined by convection and diffusion only; this leads to lower drug concentrations in the SES than in the case where SMCs are present in the SES and drug reaction with these SMCs needs to be taken into account. Given that typical P-DES in clinical use today are loaded with drug concentrations that are considerably higher than the 10μg cm^−2^ limit identified here, our results shed light onto a potential reason for the poor re-endothelialization of P-DES reported in some studies [69, 70]. Especially notable also is the observation of focal cell necrosis close to stent struts associated with high-load P-DES, which is consistent with the present results [66, 71].

Based on the present findings, we propose a number of recommendations for improved drug delivery strategies from P-DES. The first recommendation would be to lower the initial drug load by an order of magnitude and to shift the designs to slower release kinetics on the order of several months or even a year. The combination of the wide therapeutic window of paclitaxel and its long retention properties ensures sufficient efficacy in the media while the slow release of the drug precludes adverse concentrations at the endothelial surface even when SMCs are present in the SES.

The long retention of paclitaxel in the arterial wall also provides a second possible delivery strategy: quick unloading of the drug within hours thereby flooding the entire wall with paclitaxel and then letting arterial wall drug kinetics do the rest. This strategy is largely similar to the idea of a drug-eluting balloon. If administered at the right concentration and at the right time frame, the binding process takes up as much drug as is required to provide effective drug concentration levels in the entire therapeutic domain for weeks. The initial drug concentration spike is short-lived and should pose no significant problems from the standpoint of re-endothelialization because the endothelium in the vicinity of the stent struts is severely denuded during the first few days, and the extent of re-endothelialization in the first few days is limited in any case. Though promising, the outcomes associated with this strategy are expected to be quite sensitive to changes in the convective field within the arterial wall; therefore, a more accurate assessment of this field is probably needed before implementation of such a strategy. Moreover, the presence of SMCs in the SES can limit the success of this strategy. Although the SMC content in the diseased SES has been reported to be no larger than 5% of the medial SMC density [72, 73] (and see Appendix 1), in which case this quick release strategy is very promising, the SMC content is patient-dependent and thus for higher SMC densities the release strategy would need to be tailored to the individual patient.

A third possibility to address the difficulties associated with paclitaxel is to position the drug onto the stent in such a manner that it is as far away as possible from the endothelial surface. An optimization performed with our model where only the abluminal half of the stent polymeric coating contained drug (as is done for example on the BioMatrix stent by Biosensors) demonstrated, however, that this is not sufficient (data not shown). More elaborate designs, like drug-filled stents where the drug reservoir is inside of the stent body and the drug is released directly into the arterial wall via small holes (currently developed by Medtronic) or stents where small drug patches are applied only to the very abluminal surface of the stent body (similar to the JACTAX HD stent by Boston Scientific) appear to be more promising.

### Sirolimus-eluting stents require zero-order release kinetics due to sirolimus’ short retention in the arterial wall

Aside from the differences in their biological mode of action [7], the most significant difference between sirolimus and paclitaxel is the factor of 20 that separates their time scales of unbinding (Table 1). Despite sirolimus’ very high tissue affinity (its lipophilicity is approximately three times higher than paclitaxel), its short retention in the arterial wall requires a constant supply of fresh drug from the stent and renders the design of a S-DES with quasi-bolus release kinetics unfeasible. This sirolimus-specific feature has been reported in the literature [74]. The recent redesign of the zotarolimus-eluting Endeavour Resolute (Medtronic) stent (zotarolimus is a derivative of sirolimus) is another example highlighting this requirement: the release time was increased from 2 weeks in the initial design to 4 months due to the poor restenosis outcome of the original design [26, 75].

Time scale restriction aside, it should be noted that the kinetic properties of sirolimus make it a desirable drug for the design of DES: the high lipophilicity renders transport in the arterial wall largely independent of changes in the convective field and thus predictable and robust. Furthermore, initial sirolimus concentration in the stent polymer can be used to tune the concentration profile to the requirements of the arterial wall. Additionally, cytotoxicity is less of an issue for this drug due to its cytostatic mode of action. On the other hand, the lipophilicity of sirolimus also leads to more pronounced concentration peaks close to the stent struts which might compromise tissue integrity and explain the increased rate of stent strut malapposition observed with S-DES [76].

### Paclitaxel- and sirolimus-eluting stents with zero-order release kinetics lead to a similar shape of the cost function

Comparing the contour plots of the cost function for paclitaxel and sirolimus, we can identify large similarities between the two drugs in the zero-order release kinetics region. In this regime, the release from the stent coating is quasi-steady and the drug concentration at the stent surface remains nearly constant over the entire period considered. The time scales of transport and reaction in the arterial wall are all significantly faster than the release time scale so we can assume that a quasi-steady state is established in the arterial wall. For the case of constant surface concentration or equivalent constant surface flux, strongly lipophilic drugs (like paclitaxel and sirolimus) have very similar transport dynamics in the arterial wall that can be categorized depending on whether or not the applied surface concentration/flux exceeds a well-defined threshold [33].

In the case of *above-threshold* drug supply, i.e. when the drug concentration in the arterial wall exceeds the binding capacity of the arterial wall, drug transport is increased since significant amounts of drug are now in the mobile unbound state. In the case of *sub-threshold* drug supply, drug concentration in the arterial wall is below the binding capacity of the arterial wall, and drug transport is considerably decreased since the drug is now mostly in the bound and thus immobile state. For paclitaxel and sirolimus, this threshold is on the same order of magnitude as their respective binding capacities. The cost function requires that drug concentration throughout the arterial wall remain below the toxic concentration threshold, which in the case of P-DES and S-DES is below the respective binding capacities. Thus, by design of our cost function, the optimal slow-release P-DES and S-DES both fall into the sub-threshold category, hence explaining the similarity of the resulting cost function surfaces in the region of zero-order release kinetics, where transport dynamics are similar for both drugs.

### Paclitaxel vs. sirolimus: a settled debate?

About a decade ago, the FDA approved the first two DES: one eluting sirolimus (Cypher stent by Cordis) and the other eluting paclitaxel (Taxus stent by Boston Scientific). When we look at the current generation of DES that are either commercially available or at the clinical research stage, the initial 50:50 split has shifted significantly in favor of sirolimus and its derivatives to the point where P-DES appear almost “exotic” [77, 78]. This trend appears to be driven by clinical evidence which often ranks first- and second-generation S-DES ahead of their P-DES counterparts [1, 3, 79–81]. Another explanation may be the robustness of sirolimus alluded to above. However, our results offer a potentially different perspective on this issue: the applied concentrations and associated release kinetics in first-generation P-DES might just have been unsuited for the kinetics of paclitaxel; note that even though for both drugs the commercial release strategies lead to a potential inhibition of EC migration and proliferation, in the case of paclitaxel, but not in the case of sirolimus, toxic concentration levels in the media are also reached. Other numerical studies (see [11] or [45]) and several experimental studies [23, 66, 70, 71] point in a similar direction.

Consistent with our results, the one-dimensional numerical simulations [45] predict that initial polymer concentrations that are generally considered quite low can be sufficient to cause toxic concentration levels in the media and that for commercial concentration levels of 100 mol m^−3^, toxicity is reached within hours of implantation. Comparing the P-DES at four different drug loads (42 μg, 20.2 μg, 8.6 μg and 1.5 μg) in a rabbit model [71] showed an increased inflammatory response at the two higher loads compared to the lower loads. The inflammatory response was attributed to local arterial toxicity of paclitaxel. It should be noted that the 42 μg drug load is close to the load of the commercial Taxus stent. Not surprisingly, implanting even higher load P-DES (200 μg cm^−2^) in a pig model [70] also revealed high levels of arterial inflammation leading to adverse structural changes of the arterial wall. Another minipig study [66] comparing three P-DES with 430 μg cm^−2^, 145 μg cm^−2^ (close to the Taxus stent) and 72 μg cm^−2^ drug loads and reported toxic concentration levels in the arterial wall for the high load stents. Finally, proliferation assays on human coronary artery SMCs [23] revealed that paclitaxel loads four to seven times lower than found on Taxus stents can be sufficient to inhibit cell proliferation and that a prolonged release strategy of 3 months can be beneficial in terms of arterial wall toxicity, despite being less efficacious in the first few days after implantation. Our optimal release strategy results (Fig 7A) are in excellent agreement with the trends of all these experimental studies. Finally, the recent success of paclitaxel-coated balloons [74, 82–84] indicates a level of incompletely tapped potential for paclitaxel. Our results demonstrating the effectiveness of a very rapid release strategy in the case of paclitaxel provide a potential explanation for the success of paclitaxel-coated balloons.

In the present work, we have modeled diseased arteries by incorporating SMCs in the SES. The results have demonstrated that adding SMCs to the SES shifts the endothelial toxicity contour line towards lower stent loading concentrations since bound drug is now retained in the SES. Because drug efficacy in the media is unaffected by the presence of SMCs in the SES, the performance of a particular drug release strategy becomes a competition between the retention of the drug in the SES and in the media, where the first has an adverse effect on stent performance and the latter a beneficial effect. Therefore, the combination of a high Pe-number (which leads to faster drug wash-out from the SES) and a long retention time (which provides higher drug accumulation in the media) render paclitaxel a potentially very interesting drug for use in DES not only because of the possibility of a fast-release administration mode but also because it provides greater versatility (compared to sirolimus) in light of the variability in the extent of SMC content in the SES.

## Appendix

### Appendix 1. Estimating smooth muscle cell content in the subendothelial space in an atherosclerotic artery

*ξ* represents the ratio of the volume fraction of smooth muscle cells (SMCs) in the subendothelial space (SES) *χ*_SMC,ses_ to the volume fraction of SMCs in the media *χ*_SMC,m_:

$$\begin{array}{c} \xi =\frac{\chi_{\text{SMC},\text{ses}}}{\chi_{\text{SMC},m}}\;. \end{array}$$ (10)

The volume fraction of SMCs in layer *j* is the volume of SMCs divided by the total tissue volume of a sample,

$$\begin{array}{c} \xi_{\text{SMC},\text{ses}}=\frac{n_{j}V_{\text{SMC}}}{V_{\text{sample}}} \end{array}$$ (11)

where *n*_*j*_ is the number of cells in a sample volume *V*_sample_ and thus,

$$\begin{array}{c} \xi =\frac{n_{\text{ses}}}{n_{m}}\;. \end{array}$$ (12)

[73] determined the medial SMC count per mm^2^ in atherosclerotic human abdominal aortic tissue as $\approx 2016\;\frac{\text{cells}}{mm^{2}}$ and [72] measured the SMC cell density in human atherosclerotic plaques as $\approx 183\;\frac{\text{cells}}{mm^{2}}$. Assuming an isotropic distribution of SMCs, we can estimate

$$\begin{array}{c} \xi \approx \frac{{\left(\text{cells}/\text{area}\right)}_{\text{ses}}^{3/2}}{{\left(\text{cells}/\text{area}\right)}_{m}^{3/2}}\approx 3\%\;. \end{array}$$ (13)

### Appendix 2. Changes in the transport equation due to smooth muscle cell presence

Following the approach in [37], averaging the transport equation in the subendothelial space (SES) (Eq 1 in the text) with smooth muscle cells (SMCs) yields the following expression for the lag coefficient:

$$\begin{array}{c} \Lambda_{\text{SES}}=\frac{\Lambda_{f,\text{ses}}}{1-\chi_{\text{SMC},\text{ses}}} \end{array}$$ (14)

where Λ_f,ses_ is the lag coefficient based on fiber matrix theory only without SMCs as determined in [11]. For sirolimus and paclitaxel, this value is Λ_f,ses_ = 1.02. Averaging Eq 1 in the text also produces a new effective diffusivity in the SES,

$$\begin{array}{c} {\vec{D}}_{\text{SES}}=\frac{{\vec{D}}_{f,\text{eff},\text{SES}}}{\left(1-\chi_{\text{SMC,SES}}\right)f\left(\chi_{\text{SMC,SES}}\right)} \end{array}$$ (15)

where

$$\begin{array}{ccc} f(\chi_{\text{SMC},\text{ses}}) & = & \frac{2}{\sqrt{1-\frac{4}{\pi }\chi_{\text{SMC},\text{ses}}}}(\arctan\frac{1-\sqrt{\frac{4}{\pi }\chi_{\text{SMC},\text{ses}}}}{\sqrt{1-\frac{4}{\pi }\chi_{\text{SMC},\text{ses}}}}+\arctan\frac{\sqrt{\frac{4}{\pi }\chi_{\text{SMC},\text{ses}}}}{\sqrt{1-\frac{4}{\pi }\chi_{\text{SMC},\text{ses}}}})\\ & & -\frac{\pi }{2}+1-\frac{4}{\pi }\chi_{\text{SMC},\text{ses}} \end{array}$$ (16)

and ${\vec{D}}_{f,\text{eff},\text{SES}}$ is the effective diffusivity coefficient from fiber matrix theory in the SES as determined in [11], with a value of ${\vec{D}}_{\text{f,eff,SES}}^{\text{PAX}}=1.7\times 10^{-11}{\text{m}}^{2}{\text{s}}^{-1}$ for paclitaxel and ${\vec{D}}_{\text{f,eff,SES}}^{\text{SIR}}=1.67\times 10^{-11}{\text{m}}^{2}{\text{s}}^{-1}$ for sirolimus.
